# Genetic Architecture and Genomic Prediction of Cooking Time in Common Bean (*Phaseolus vulgaris* L.)

**DOI:** 10.3389/fpls.2020.622213

**Published:** 2021-02-11

**Authors:** Santiago Diaz, Daniel Ariza-Suarez, Raisa Ramdeen, Johan Aparicio, Nirmala Arunachalam, Carlos Hernandez, Harold Diaz, Henry Ruiz, Hans-Peter Piepho, Bodo Raatz

**Affiliations:** ^1^Bean Program, Agrobiodiversity Area, International Center for Tropical Agriculture (CIAT), Cali, Colombia; ^2^Institute of Crop Science, University of Hohenheim, Hohenheim, Germany; ^3^Departamento de Agronomía, Facultad de Ciencias Agrarias, Universidad Nacional de Colombia, Bogotá, Colombia; ^4^Instituto Tecnológico de Durango, Victoria de Durango, Mexico

**Keywords:** genome-wide association mapping (GWAS), QTL, cooking, prediction, bean

## Abstract

Cooking time of the common bean is an important trait for consumer preference, with implications for nutrition, health, and environment. For efficient germplasm improvement, breeders need more information on the genetics to identify fast cooking sources with good agronomic properties and molecular breeding tools. In this study, we investigated a broad genetic variation among tropical germplasm from both Andean and Mesoamerican genepools. Four populations were evaluated for cooking time (CKT), water absorption capacity (WAC), and seed weight (SdW): a bi-parental RIL population (DxG), an eight-parental Mesoamerican MAGIC population, an Andean (VEF), and a Mesoamerican (MIP) breeding line panel. A total of 922 lines were evaluated in this study. Significant genetic variation was found in all populations with high heritabilities, ranging from 0.64 to 0.89 for CKT. CKT was related to the color of the seed coat, with the white colored seeds being the ones that cooked the fastest. Marker trait associations were investigated by QTL analysis and GWAS, resulting in the identification of 10 QTL. In populations with Andean germplasm, an inverse correlation of CKT and WAC, and also a QTL on Pv03 that inversely controls CKT and WAC (CKT3.2/WAC3.1) were observed. WAC7.1 was found in both Mesoamerican populations. QTL only explained a small part of the variance, and phenotypic distributions support a more quantitative mode of inheritance. For this reason, we evaluated how genomic prediction (GP) models can capture the genetic variation. GP accuracies for CKT varied, ranging from good results for the MAGIC population (0.55) to lower accuracies in the MIP panel (0.22). The phenotypic characterization of parental material will allow for the cooking time trait to be implemented in the active germplasm improvement programs. Molecular breeding tools can be developed to employ marker-assisted selection or genomic selection, which looks to be a promising tool in some populations to increase the efficiency of breeding activities.

## Introduction

Common bean (*Phaseolus vulgaris* L.) is one of the most important cultivated grain legumes and is consumed by millions of people worldwide, particularly in developing countries in the tropics (Broughton et al., 2003). Bean is one of the crops targeted for biofortification because it is a rich and relatively inexpensive source of proteins and micronutrients such as iron and zinc (Beebe, 2012). The common bean is organized in two genetically differentiated genepools: The Mesoamerican and the Andean genepools, which diverged from a common ancestral wild population more than 100,000 years ago. In these genepools, independent domestication events resulted in landraces with diverse attributes (Schmutz et al., 2014).

Grains that cook faster are preferable due to the lower time taken for meal preparation usually carried out by women, which would allow them to pursue other tasks (Ribeiro et al., 2014). Another important issue is the economy of energy use. The energy for cooking represents about 90% of all household energy consumption in developing countries using wood as a major fuel source. When this wood is burned, it contributes to high levels of local air pollution (De et al., 2013). Moreover, fuelwood in urban areas is costly, while collection in rural regions traditionally is a task carried out by women and children and may be time consuming and associated with risks. This has a direct impact on the food chosen by women to cook in those countries (Masangano and Miles, 2004).

From a nutritional perspective, cooking time is important because it affects the content of phytochemicals with antinutritional effects (ElMaki et al., 2007; Yasmin et al., 2008; Wang et al., 2010). Prolonged cooking times were reported to reduce and degrade nutrients important for the human diet (Pujolà et al., 2007; Shiga et al., 2009; Chinedum et al., 2018). Research also revealed that fast cooking beans retain more minerals and proteins after being fully cooked compared to other slow cooking beans, showing the higher nutritional value of fast cooking beans (Wiesinger et al., 2016, 2018). Therefore, a shorter cooking time can have a positive impact on consumers, freeing up time, as well as improving nutrition, health, and economy, especially in areas where beans are consumed as a primary source of protein. Several methods have been used to evaluate cooking time for beans. At present, the time-consuming Mattson cooker method is mostly used (Carvalho et al., 2017).

Several factors affecting cooking time have been studied, such as characteristics and composition of seeds, growing location, and storage conditions (Arruda et al., 2012; Wani et al., 2017). However, the genetic architecture of cooking time is less understood. Some studies indicate this is a trait controlled by few genes and presents relatively high heritability values (Elia et al., 1997; Jacinto-Hernandez et al., 2003; Arns et al., 2018). Other studies report high genetic variability of the trait, and several genomic regions may be involved in its genetic control (Vasconcelos et al., 2012; Cichy et al., 2015; Berry et al., 2020).

Different strategies have been used for genetic mapping. In linkage mapping, a bi-parental population is utilized to identify the genomic regions that segregate with a trait, but this strategy is usually low in resolution since only two alleles per locus are analyzed, and genetic recombination is limited (Islam et al., 2016). Genome-wide association mapping (GWAS) directly identifies marker-trait associations in natural or constructed populations based on linkage disequilibrium (LD). This strategy does not demand generating populations and uses the historical genetic recombinations available in panels. However, the population structure can produce a high LD between non-linked markers (Klasen et al., 2016). Lately, a multi-parent advanced generation intercross (MAGIC) strategy has been proposed to increase precision and resolution. In MAGIC populations, QTLs and marker-trait associations can be detected due to the increased level of recombination, and present more phenotypic and genetic variability than biparental populations (Bandillo et al., 2013). Therefore, combining GWAS and QTL analysis not only avoids the false positives from associated loci due to high LD but also facilitates fine mapping of a target region with a large QTL interval (He et al., 2017). However, certain traits display a quantitative mode of inheritance, are governed by many different QTL of small effects across the genome, and are highly influenced by genotype-by-environment interactions. All these factors define the complexity of these traits, and elucidating the underlying genetic basis proves to be a difficult task. Genomic prediction (GP) is a recent promising tool for plant breeding for phenotype prediction based on genomic estimated breeding values (GEBV) estimated from information on genome-wide markers (Crossa et al., 2017). GPs are suitable for quantitative traits controlled by many genes. This method has a high potential, mainly when phenotyping is costly and laborious (Spindel et al., 2015; Minamikawa et al., 2017; Muleta et al., 2017).

The objective of this study was to investigate the genetic architecture of cooking time in beans in a bi-parental population, germplasm collections, and a MAGIC population. QTL and GWAS analysis were combined to identify genomic regions involved in the trait. Genomic prediction methods are evaluated to assess the predictive accuracy for genomic prediction models for cooking time.

## Materials and Methods

### Germplasm

In this study we used four different populations of common bean to elucidate the genetic architecture of cooking time: (1) A bi-parental population (DxG) previously described by Blair et al. (2003) and Galeano et al. (2009), which consists of 87 recombinant inbred lines (RIL). This population was obtained by crossing DOR364, an improved Mesoamerican line from the International Center for Tropical Agriculture (CIAT), and the Andean accession G19833, a landrace from Peru. The DxG population was advanced by a modified single-seed descent (SSD) to F_9:11_ generation. (2) A multiparent advanced-generation intercross (MAGIC) population previously described by Diaz et al. (2020). This population used a set of eight Mesoamerican breeding lines from CIAT as founders. The details of the MAGIC crossing scheme are presented in Supplementary Figure 1. This population contained 636 F_4:6_ RILs generated by the SSD method. (3) The “Vivero Equipo Frijol” (VEF) panel, previously described by Keller et al. (2020) is composed of 380 Andean breeding lines. (4) The “Mesoamerican Introgression Panel” (MIP) consists of 222 breeding lines of the Mesoamerican gene-pool. This panel was assembled to study to which extent interspecific introgressions from other species of *Phaseolus* (*P. acutifolious*, *P. dumosus*, *and P. coccineus*) have been introduced by the Mesoamerican breeding program over recent decades. Therefore, the panel consists of recent breeding lines as well as available ancestors and initial interspecific introgression pre-breeding lines.

### Field Trials

The field trials of all four populations were planted at the CIAT Palmira experimental field station (Colombia, altitude of 1,000 m.a.s.l., latitude 3°32′N and longitude 76°18′W). The experimental unit in these trials were row plots of 2.22 m^2^ laid out for each replicate of each line. The DxG population and its parental lines were established in the field following a randomized complete block design with three replicates for each RIL in 2011. The MAGIC population and its eight founders were planted in 2013 in an alpha-lattice incomplete-block design with three replicates as described by Diaz et al. (2020). A subset of 223 MAGIC lines and its eight founders were phenotyped for cooking time. The VEF panel was planted in 2017 with an alpha-lattice incomplete block experimental design of three replicates as described by Keller et al. (2020). The MIP panel was grown in 2018 following an alpha-lattice incomplete block design with three replicates. In this panel, 66 lines were phenotyped for cooking time with three replicates, while only one replicate was phenotyped of the remaining lines. In all trials, seed was harvested manually by plot upon maturity (120–140 days after sowing). The collected seed was cleaned to remove debris and damaged seed, and dried until reaching an average moisture content of 10–14% (determined with a moisture meter MT-16 Grain Moisture Tester, AgraTronix, United States). The MAGIC population, VEF panel, and MIP panel were stored at controlled temperature (4°C) and low humidity (< 30%) in a cold room. The storage conditions for the DxG population were not as optimal as they were for the other three populations (Kinyanjui et al., 2017). The DxG population was stored in a room without controlled storage conditions (22–32°C temperature room and high humidity > 60%). This may cause the cases of hard-to-cook (HTC) phenotypes in that population.

### Cooking Time and Water Absorption Capacity

The water absorption capacity (WAC) was measured using the method described by Deshpande and Cheryan (1986): a subsample of 28 seeds per replication was weighed (SdW) and soaked in distilled water for 12 h at room temperature (25°C). After that, seeds were drained, blotted dry, and weighed again to determine the water absorption during soaking with the formula:

$$WAC\left(\%\right)=\frac{\begin{array}{c} S\,d\,W\,a\,f\,t\,e\,r\,s\,o\,a\,k\,i\,n\,g\,\left(g\right)\\ -S\,d\,W\,b\,e\,f\,o\,r\,e\,s\,o\,a\,k\,i\,n\,g\,\left(g\right) \end{array}}{\begin{array}{c} S\,d\,W\,b\,e\,f\,o\,r\,e\,s\,o\,a\,k\,i\,n\,g\,\left(g\right) \end{array}}\times 100$$

Cooking time (CKT) was measured on 24 seeds with the Mattson pindrop cooker (Customized Machining and Hydraulics Co., Winnipeg, MB, Canada. *Modified at* CIAT—*more information below*). Each soaked seed was placed in a well of the plate. On top of each seed a 90-g piercing pin was set down, and the Mattson device was placed in boiling distilled water (> 98°C). For each seed, the time (expressed in minutes) that it took to be completely pierced by the pin was recorded (Wang and Daun, 2005). In this study, CKT was defined for each sample as the 80^th^ percentile of the evaluated seed per experiment (usually 24 seeds). The time between harvest to the evaluation moment was more than 2 years for the DxG and the MAGIC population, and less than 6 months for the VEF panel and MIP panel.

### Hardware and Software Design for Measuring Cooking Time

The Mattson cooker was modified to become partially automated using an embedded system for taking data from each seed individually. The system uses a custom-made printed circuit board assembly with 24 installed micro-switches that detect if any of the 90-g stainless steel piercing pins pierce the bean. A ribbon cable connects the plate to a Udoo micro computer system harboring a router for Wi-Fi communication. Furthermore, a PT100 sensor was added to allow monitoring of the temperature throughout the experiment. Finally, a web application was developed to monitor and control wirelessly the process on any computer or mobile device (for *more information, see Supplementary Document 1*).

### Data Analysis

Statistical analysis of the phenotypic data was conducted with statistical software R (v3.3.2). A Grubbs test (Grubbs, 1950) was used to identify and remove outliers in each dataset of 24 time values of each pin in a given experiment using the R package “outliers” (v0.14) (Komsta, 2011). Best linear unbiased estimators and predictors (BLUEs/BLUPs) were calculated for CKT and WAC using the “lme4” Package (Bates et al., 2015). The data from each trial were modeled using the following formula:

(1) $$y_{m\,i\,j\,k}=\mu +G_{m}+M_{i}+R_{j}+{\left(R\,B\right)}_{j\,k}+\varepsilon_{m\,i\,j\,k}$$

where *y* is a vector with the phenotypic responses, μ is the overall intercept, *G*_*m*_ is the effect of the *m*^th^ genotype, *M*_*i*_is the effect of the *i*^th^ machine, *R*_*j*_ is the effect of the *j*^th^ replicate, (*RB*)_*jk*_ is the effect of the k^th^ block nested within the *j*^th^ replicate (which was only included for alpha designs), and ε_*mijk*_ is the error term corresponding to *y*_*mijk*_. In this model, the terms *M*_*i*_, *R*_*j*_, and (*RB*)_*jk*_ were treated as random effects. The *G*_*m*_ term effects were treated either as fixed (to calculate BLUEs) or random (to get an estimate of the genetic variance and calculate BLUPs). We assumed that every random term *u* and the residual ε adjusts to a normal distribution with mean 0 and independent variances $u∼N\,\left(0,\sigma_{u}^{2}\,I\right)$ and ε∼N⁢(0,σε⁢I2).

To determine the proportion of the genetic variance controlling CKT and WAC for each population, broad-sense heritability (H^2^) estimates were calculated using the method proposed by Cullis et al. (2006). Trait H^2^ estimates were computed using the equation below:

(2) $$H^{2}=1-\frac{\upsilon_{B\,L\,U\,P}}{2\,\sigma_{g\,e\,n\,o\,t\,y\,p\,e}^{2}}$$

where υ_*BLUP*_ is the mean variance of a difference of two BLUPs of genotypic effects, and $\sigma_{g\,e\,n\,o\,t\,y\,p\,e}^{2}$ is the genetic variance. The phenotypic correlation between traits of interest was expressed as Pearson’s correlation coefficients among BLUEs, and their significance was tested using a two-tailed *t*-test.

The differences between seed color groups with regard to CKT were modeled as:

(3) $$y_{i\,j}=\mu +c_{i}+\varepsilon_{i\,j}$$

where *y*_*ij*_ is a vector of BLUEs obtained from equation 1, μ is the intercept, *c*_*i*_ is the effect of the *i*^th^ color group, ε_*ij*_ is the error term, and we assumed ε∼N⁢(0,σε⁢I2).

### Genotyping

The development of molecular markers and construction of a genetic linkage map for the DxG population was described in detail in previous studies (Blair et al., 2003, 2006, 2008; Galeano et al., 2009) where 561 markers were mapped to 11 linkage groups with a 2,731 cM distance. The map was developed with the Kosambi mapping function using the MapDisto Software (v1.7) (Lorieux, 2012). The graphic visualization of the DxG genetic map was created in MapChart (v2.32) (Voorrips, 2002).

Genotyping of the MAGIC population and its founders (629 RIL + 8 lines), the VEF panel (330 lines), the MIP panel (210 lines), and each founder genotype of the DxG population was obtained using the *Ape*KI-based genotyping-by-sequencing (GBS) protocol (Elshire et al., 2011), as previously described (Perea et al., 2016; Gil et al., 2019; Keller et al., 2020; Diaz et al., 2020). Briefly, DNA was extracted using the urea buffer-based DNA extraction midi prep protocol. GBS libraries were sequenced at the HudsonAlpha Institute for Biotechnology^1^ and the Institute of Biotechnology of Cornell University^2^. Each 96-well plate was sequenced in one lane of an Illumina HiSeq platform device using single-end or paired-end reads ranging between 100 and 150 bp.

The mapping and variant calling processes for GBS reads is described in detail by Perea et al. (2016), Gil et al. (2019), and Keller et al. (2020). In brief, the GBS reads were demultiplexed using NGSEP (v3.3.2) (Tello et al., 2019). Adapters and low-quality bases from the raw sequencing data were trimmed using Trimmomatic (v0.36) (Bolger et al., 2014), and reads were aligned to the reference genome of *P. vulgaris* G19833 v2.1. (Schmutz et al., 2014) using Bowtie2 (v2.2.30) (Langmead and Salzberg, 2012) with default parameters. The variant calling process was performed using NGSEP following recommended parameters for GBS data (Perea et al., 2016). The resulting genotype matrix was filtered to remove genotype calls with quality below 40, remove markers with more than 6% heterozygote calls, minor allele frequency (MAF) below 0.02, and remove markers in repetitive regions of the reference genome (Lobaton et al., 2018). Markers were selected so that the overall missing data rate in the matrix does not surpass 20%. The process to construct a genotype matrix was carried out for each population separately. The final matrices contained 17,654 SNP markers for the MAGIC population, 9,421 SNP markers for the VEF panel, and 26,500 SNP markers for the MIP panel. These matrices were used thereafter for the GWAS analysis (with an independent principal components analysis and kinship calculation for each population) and for single-trait assessment of prediction ability. In addition, the same SNP calling and matrix filtering processes were repeated combining the sequencing data of the DxG founder genotypes and all three populations (MAGIC, VEF, and MIP). The resulting joint genotype matrix contained 17,508 SNP markers and was used to assess the population structure by performing a principal component analysis (PCA) using GAPIT (v3.0) (Tang et al., 2016) and to perform the second case of the cross-prediction scenario (detailed in section “Genomic Prediction Models”).

The construction of the genetic map of the MAGIC population was described in detail by Diaz et al. (2020) using the method for haplotype prediction reported in an *Arabidopsis thaliana* MAGIC population (Kover et al., 2009). Briefly, the inferred haplotypes were used to transform the GBS matrix with allele information into a matrix with founders’ source information for each marker. The marker set in this matrix was reduced by a binning procedure based on their recombination frequency, generating a subset of 5,738 non-redundant markers. This filtered matrix was used to construct the genetic map with the Kosambi mapping function using the integrated genetic analysis software for multi-parental pure-line populations (GAPL) (v2.1) (Zhang et al., 2019) (*for more details about the methods applied for each population, see Supplementary Figure 2*).

### QTL and Genome-Wide Association Mapping Analysis

QTL analysis for the DxG and MAGIC populations was conducted using the genetic maps of each population and the calculated BLUEs. Detection of QTLs and estimation of genetic parameters for CKT and WAC were performed using the composite interval mapping (CIM) procedure of the software IciMapping (v4.1) (Meng et al., 2015) with 10 cM windows and a sliding parameter of 1 cM for DxG population. Significant QTL were considered by defining the logarithm of odds (LOD) score at a genome-wide type I error rate of a α = 0.05 after 1,000 permutation tests for each trait, obtaining a significance threshold of 3.24 LOD for the DxG population. For the MAGIC population, the composite interval mapping was carried out with the procedure for additive effects (ICIM-ADD) of the software GAPL (v1.2) (Zhang et al., 2019), employing the forward and backward regression model, with 5 cM windows and a sliding parameter of 0.5 cM. Significant QTL were defined at 6.68 LOD for the MAGIC population following the same permutation tests defined for the DxG population.

The association analysis was carried out in the MAGIC population, VEF, and MIP panels using the R package GAPIT (v3.0) (Tang et al., 2016), providing the genotypic matrix that was produced for each population independently, and their corresponding BLUEs. The association analysis was conducted using a mixed linear model (MLM) approach. This model accounts for population structure using the top five principal components (described previously) as fixed effects. It also accounts for random polygenic effects with a kinship matrix as variance–covariance structure, calculated using the method proposed by VanRaden (2008) implemented in the GAPIT package. Significant associations were defined when the *p* value was equal to or smaller than the Bonferroni-corrected threshold (2.38 × 10^–6^ for MAGIC population, 5.30 × 10^–6^ for VEF panel, and 1.88 × 10^–6^ for MIP panel) (Johnson et al., 2010). Manhattan and QQ plot graphics were made using the qqman R package (Turner, 2018).

### Genomic Prediction Models

A single-trait assessment of prediction ability was performed for each population individually using the R package BGLR (v1.0.8) (Pérez and De Los Campos, 2014), with 10,000 iterations, using the first 2,000 for burn-in and default parameters. In each case, 70% of the individuals in the population were assigned to the training set, while the remaining 30% were assigned to the validation set, following the results from Keller et al. (2020). This random assignment was repeated 100 times for each population. Prediction ability (PA) was defined as the Pearson correlation coefficient (*r*) between the observed (true breeding values or TBVs) and the predicted values (genomic estimated breeding values or GEBVs) of the validation set. Different priors for parametric regressions on the SNP markers were tested, including the Bayesian ridge regression (BayesRR), BayesA, BayesB, BayesC, Bayesian Lasso (BLasso), and a GBLUP model. All these priors are based on additive effects models. In addition, Bayesian reproducing kernel Hilbert spaces regression (RKHS) was tested fitting a single Gaussian kernel model in the (average) squared-Euclidean distance between genotypes, as defined by Pérez and De Los Campos (2014), with a fixed bandwidth parameter *h* = 0.5. The RKHS model is a semi-parametric approach that incorporates additive and non-additive effects.

A cross-prediction scenario was tested between and within populations and traits, using the RKHS model described above. Unlike the single-trait assessment of genomic prediction, different datasets were used for training and validation in this case. This scenario was divided in two separate cases. The first consisted in using the BLUEs of CKT, WAC, or SdW from a single population to predict every other trait in the same population. In total, 18 different combinations of training-validation datasets were obtained for this first case. For each combination, a similar cross validation process used 70% of the individuals from the training set of BLUEs to train the model. The remaining 30% of individuals from the validation set of BLUEs were used to calculate the PA between observed (TBVs) and predicted values (GEBVs). The random 70:30 assignment was repeated 100 times. The second case used the BLUEs from a given trait-population to train the model, and then it was used to predict every other trait in a different population, producing 54 different combinations of training-validation datasets. In this case, no cross validation was done, performing a single training and validation step. This second case of cross-prediction scenario resembles a more realistic breeding context.

## Results

### Phenotypic Variation for Cooking Time, Water Absorption Capacity, and Seed Weight in Four Populations

Cooking time, WAC, and SdW were evaluated in four different populations incorporating a large genetic variability: The DxG population (bi-parental Andean × Mesoamerican inter-genepool RIL population), the MAGIC population (eight-parental Mesoamerican population), the VEF panel (Andean breeding lines), and the MIP panel (Mesoamerican breeding lines).

**FIGURE 1:**
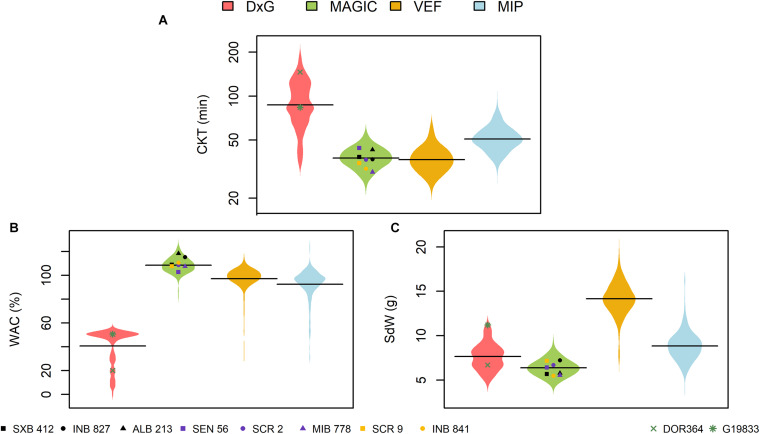
Phenotypic distributions of **(A)** cooking time (CKT), **(B)** water absorption capacity (WAC), and **(C)** 28 seed weight (SdW) in four evaluated populations of common bean. DxG is a RIL population product of the cross between DOR364 and G19833 (green points). The MAGIC population was produced by intercrossing the eight founder lines displayed as black, purple, and yellow points. The VEF and MIP panels are composed of elite breeding lines from the Andean and Mesoamerican genepools, respectively.

Significant phenotypic variability was observed in all populations for all traits (Figure 1 and Supplementary Table 1). The DxG population presented more phenotypic variability for CKT and WAC compared to the other populations (Figure 1) with the highest coefficient of variation (> 30%). The highest CKT values were found for DxG with an average of 92.9 min, while it also had the lowest WAC values, with an average of 40.7%. Furthermore, DxG displayed problems with water absorption as several samples failed to absorb significant amounts of water. These effects may be attributed to the storage conditions of the seed, as the DxG seed was stored for a longer period before evaluation. The Andean parent G19833 presented a shorter CKT (83.7 min) than the Mesoamerican parent DOR364 (146 min) (Figure 1). G19833 has larger seeds that showed higher WAC compared to DOR364 (Figure 1). The spatial variation in the field that was modeled with the *R*_*j*_ and (*RB*)_*jk*_ terms in equation (1) were not significant for any trial. Since they were accounted for as random effects terms, the model presented in equation (1) automatically drops the zero variance terms and reduces itself.

The MAGIC population, VEF, and MIP panels presented similar phenotypic variability for CKT, with the lowest average values for the VEF (37.4 min), MAGIC (38.1 min), and MIP (51.6 min) (Figure 1A). In the MAGIC population, the founder with the lowest CKT was MIB778 (30.2 min) (Figure 1A). The MAGIC population presented a higher WAC (108.5%) compared with the other populations. The line ALB213 showed the highest WAC value among the founder lines of the MAGIC population (118.2%) (Figure 1B). The large seeded Andean VEF panel had average WAC values in between the Mesoamerican MAGIC and MIP panels, and expectedly the heaviest seeds (Figures 1B,C). All the evaluated traits showed high heritabilities in all populations (Supplementary Table 1). For CKT, the heritabilities ranged from 0.64 to 0.89, while other traits had values ranging between 0.68 and 0.93, indicating good data quality for further analysis.

Correlations between the evaluated traits were somewhat inconsistent between populations (Table 1). Significant negative correlations between CKT and WAC were observed in the DxG and VEF panels, which belong (at least partially) to the Andean genepool, whereas correlations were not significant in the other two populations. The correlations between CKT and SdW were significant and positive in the case of the MAGIC population, but negative for the VEF and MIP panels. A negative significant correlation between WAC and seed size was only observed for the MIP panel. Taken together, higher water absorption is correlated to earlier CKT in half the experiments. However, we observed no general effect of seed size or genepool on CKT or WAC as the correlations with seed weight were not consistent.

**TABLE 1:** Pearson’s correlation and significance between cooking time (CKT), water absorption capacity (WAC), and 28 seed weight (SdW) of four populations (DxG population, MAGIC population, VEF panel, and MIP panel).

Population	Trait	WAC	SdW
**DxG**			
	CKT	−0.46***	–0.18
	WAC		–0.04
**MAGIC**			
	CKT	0	0.19***
	WAC		0.01
**VEF**			
	CKT	−0.28***	−0.33***
	WAC		0.13
**MIP**			
	CKT	–0.07	−0.13*
	WAC		−0.23***

Significance of correlations indicated as ***p < 0.001; **p < 0.01; *p < 0.05.

**FIGURE 2:**
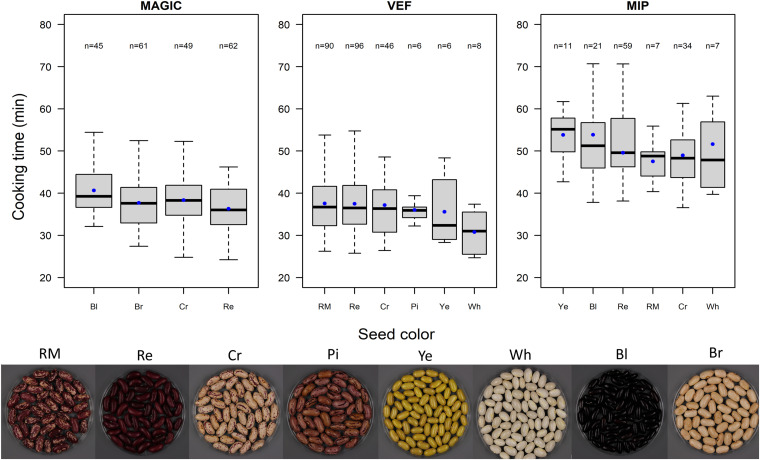
Boxplot of cooking times for the MAGIC population, VEF panel, and MIP panel grouped by seed color. Blue dots represent cooking time averages for each group. Seed color abbreviations are as follows: *RM*, red mottled; *Re*, red; *Cr*, cream; *Pi*, pink; *Ye*, yellow; *Wh*, white; *Bl*, black; and *Br* brown.

We investigated the effect of seed color on CKT. In the VEF and MIP panels, light colored beans presented faster cooking time compared to darker beans, the white seeded being the fastest cooking group (Figure 2). However, the variance component for color group in equation (3) was significant only within the MAGIC population, where the seeds with black coat presented the lowest CKT.

The top 5% of the fastest cooking lines for each population are listed in Supplementary Table 2. Overall, the fastest cooking lines (with less than 25 min cooking time) belong to the VEF (LPA_566, LPA_071, SAA_020, SAB_576, and AFR_619) and MAGIC panels (MGC_263 and MGC_330). In the DxG and MIP populations, the fastest cooking lines ranged between 31 and 39 min. Only four genotypes in the DxG population were listed in this range (DxG_80, DxG_53, DxG_22, and DxG_26), while 11 lines from the MIP panel were included in the list (SEF_070, SMR_152, SMC_223, MIB_778, SXB_412, SMC_040, SMC_143, MIB_755, SMC_228, SMN_071, and SMR_048).

### Genetic Structure of the Populations of Interest

**FIGURE 3:**
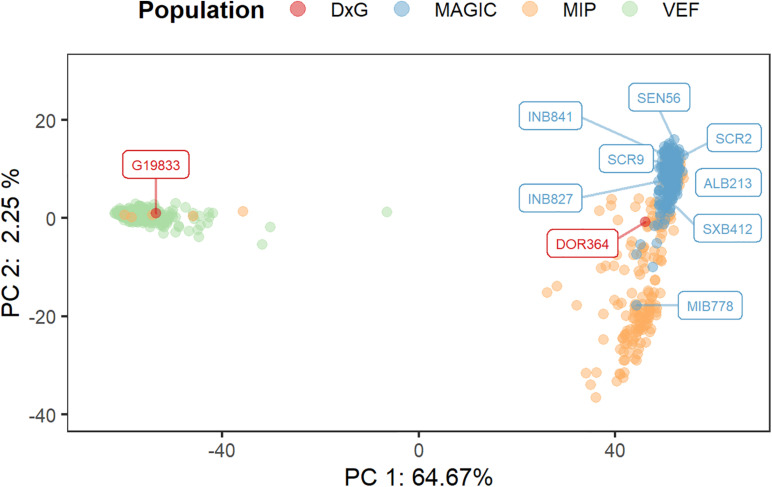
Assessment of population structure by a principal components analysis (PCA) using 17,508 SNP markers from the combined MAGIC, VEF, and MIP lines. The location of each genotype is represented by a point in the two-dimensional space defined by the eigenvectors of the first and second principal components. The founder lines of the MAGIC and DxG populations are represented by colored tags.

The sequencing data from the DxG parental lines, the MAGIC population, the VEF, and MIP panels were merged into a single matrix of 17,508 polymorphic markers that were used to assess the population structure (Figure 3). The first principal component explains a major proportion of the total variance, accounting for 64.7%. This PC separates the samples into two clearly differentiated clusters that correspond to the Andean genepool (left side) with the VEF lines and the DxG parental line G19833, and the Mesoamerican genepool (right side) comprised of the MAGIC and MIP lines, and the second DxG parental DOR364. MAGIC and most MIP lines are separated by the second PC as the majority of variation that is not explained by the genepool is found in the Mesoamerican lines. Taken together, these results indicate significant differences in the population structure between populations.

### QTL and Genome-Wide Association Mapping Analysis

To investigate the genetic basis of the CKT and WAC, QTL analyses were performed on the biparental DxG population and the multi-parental MAGIC population, and GWAS was carried out on the MAGIC, VEF, and MIP panels.

For the QTL analyses, the genetic map previously reported for DxG was used (Galeano et al., 2009) (Supplementary Figure 3). Three QTL were identified on chromosomes Pv01 and Pv03, two QTL for CKT, one of which co-located with a WAC QTL (Table 2). The identified CKT QTL account for 15.8–16.0% of the observed variance, whereas WAC3.1 explains 69.76%. The Andean parent G19833 contributes the allele for lower CKT and higher WAC, in line with the observed phenotypic correlation of both traits and the phenotypes of the parents (Table 1). CKT3.2 and WAC3.1 are located in the same position, suggesting that the same polymorphism might be affecting both traits (Supplementary Figure 4).

**TABLE 2:** QTL identified for cooking time (CKT) and water absorption capacity (WAC) in DOR364 × G19883 RIL population using Composite Interval Mapping.

QTL name	Chr.	Pos. (cM)	Physical pos. left marker (Mbp)^a^	Physical pos. right marker (Mbp)^a^	Left marker name	Right marker name	LOD	PVE (%)^b^	Add^c^	Interval position (Mbp)
*CKT1.1*	Pv01	344	50.9	51.31	Bng083	g510	3.94	15.8	13.04	0.37
*CKT3.2*	Pv03	237	51.1	52.06	Leg213	BMb590	3.67	15.97	13.88	0.92
*WAC3.1*	Pv03	237	51.1	52.06	Leg213	BMb590	26.76	69.76	–14.5	0.92

*^*a*^Physical position in Mbp.*

*^*b*^Phenotypic variation explained. ^*c*^Allelic effect by DOR364.*

The genetic map previously reported for the MAGIC population was used for QTL analyses (Diaz et al., 2020; Supplementary Table 3). Five QTL were mapped in the MAGIC population using the haplotype-based interval mapping, four QTL for CKT, and one for WAC (Table 3). In all four QTL for CKT, MIB778 (fastest cooking time founder) and INB827 contributed negative allelic effects reducing cooking time (Supplementary Table 4). CKT3.1 (∼0.5 Mbp) observed in the MAGIC population is distant from CKT3.2 identified in the DxG population (∼51 Mbp).

**TABLE 3:** QTL mapped for cooking time (CKT) and water absorption capacity (WAC) in the MAGIC population based on composite interval mapping using haplotype prediction with the procedure for additive effects (ICIM-ADD).

QTL name	Chr.	Pos. (cM)	Left marker name^a^	Right marker name	LOD	PVE (%)^b^	Interval size (Mbp)
*CKT3.1*	Pv03	1.5	Pv2.1_03_592656_G/A	Pv2.1_03_755530_G/C	6.96	7.07	0.16
*CKT4.1*	Pv04	47	Pv2.1_04_41895594_A/T	Pv2.1_04_41987047_A/T	7.03	7.60	0.09
*CKT7.1*	Pv07	63.5	Pv2.1_07_31833933_A/C	Pv2.1_07_32077935_T/C	11.39	12.18	0.24
*CKT8.1*	Pv08	77	Pv2.1_08_60150805_T/C	Pv2.1_08_60180011_A/G	9.01	9.77	0.02
*WAC11.1*	Pv11	75	Pv2.1_11_51537051_G/C	Pv2.1_11_51584833_C/G	7.96	8.69	0.04

*Information on founder haplotype effects is in Supplementary Table 4.*

*^*a*^Physical position and polymorphism of the SNP according to reference version 2.1.*

*^*b*^Phenotypic variation explained.*

**FIGURE 4:**
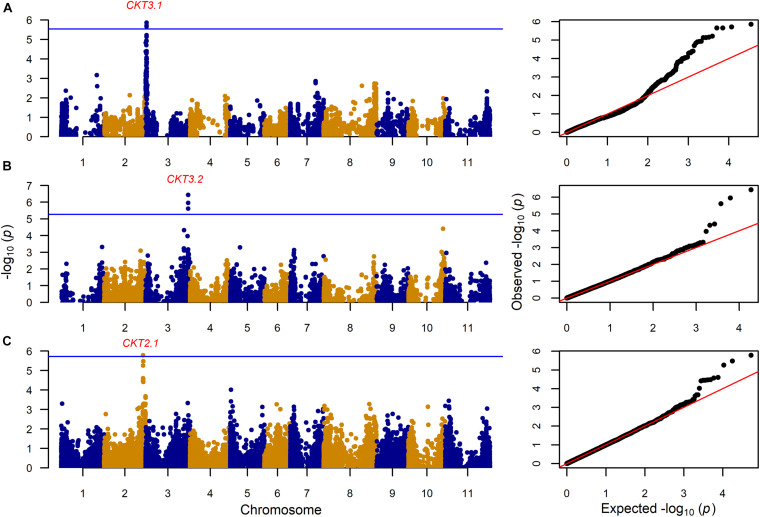
Genome wide association analysis for cooking time (CKT) showing Manhattan and QQ plot for **(A)**: 203 MAGIC RILs with 17,654 markers, **(B)** 330 VEF lines with 9,420 markers, and **(C)** 197 MIP lines with 26,500 markers. The Bonferroni correction threshold (*p* = 0.05) is presented as a blue horizontal line based on the number of markers for each population, respectively.

**FIGURE 5:**
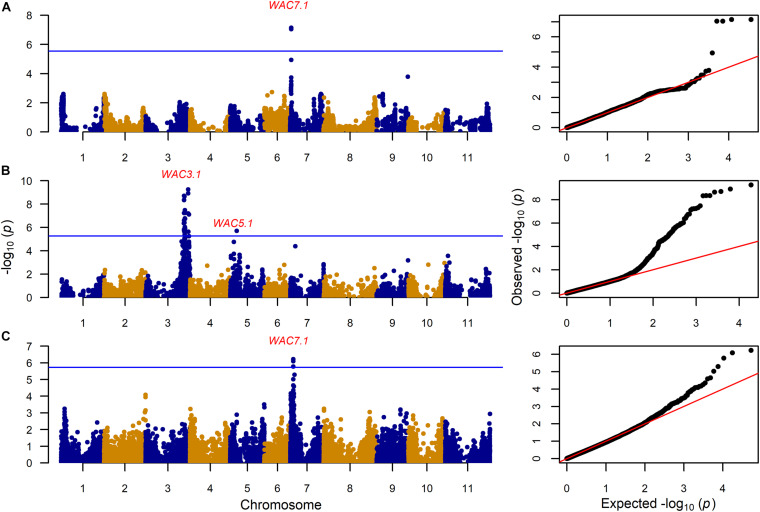
Genome wide association analyses showing Manhattan and QQ plots for water absorption capacity (WAC) for **(A)**: 203 MAGIC RILs with 17,654 markers, **(B)** 330 VEF lines with 9,420 markers, and **(C)** 193 MIP lines with 26,500 markers. The Bonferroni correction threshold (*p* = 0.05) is presented as a blue horizontal line based on the number of markers in each population, respectively.

**FIGURE 6:**
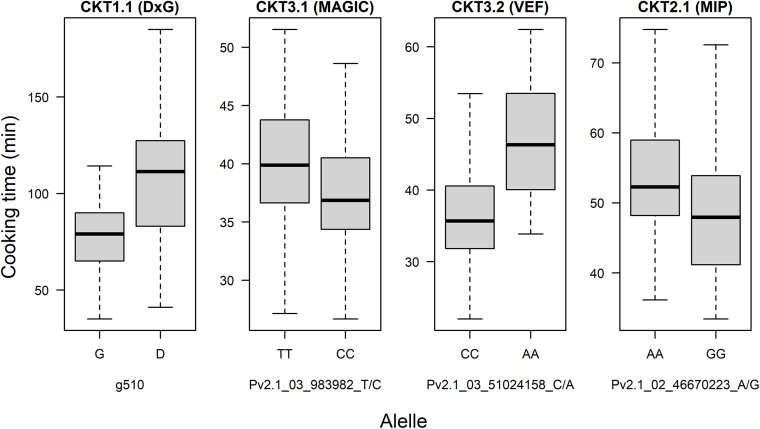
Allelic effect for the most significant marker of QTL of cooking time found in the DxG population, MAGIC population, VEF panel, and MIP panel.

GWAS on the MAGIC population, VEF, and MIP panels revealed six QTL, three for CKT and three for WAC (Table 4 and Figures 4, 5, Supplementary Tables 5,6). CKT3.2 was identified in the VEF panel on the same position as in the DxG population (Table 4 and Figure 4). CKT3.2 has the largest allelic effect on CKT (5.24 min) and explained a large part of the phenotypic variation (20%) (Figure 6). Similarly, WAC3.1, was found in the DxG and VEF panel on the same position as CKT3.2. Both QTL for CKT and WAC have the same allelic frequency (0.95) (Table 4). This is a distinctive QTL of Andean origin.

**TABLE 4:** QTL and most significant markers identified by GWAS associated with cooking time (CKT) and water absorption capacity (WAC) in MAGIC population, VEF panel, and MIP panel.

QTL name	Population	Marker	Chr.	Pos. (bp)^a^	*p* value	MAF^b^	n	R^2c^	Effect
*CKT3.1*	MAGIC	Pv2.1_03_983982_T/C	Pv03	983,982	1.39E−06	0.38	203	0.34	–1.98
*CKT3.2*	VEF	Pv2.1_03_51024158_C/A	Pv03	51,024,158	3.66E−07	0.05	330	0.20	5.24
*CKT2.1*	MIP	Pv2.1_02_46670223_A/G	Pv02	46,670,223	1.66E−06	0.46	197	0.17	–0.47
*WAC7.1*	MAGIC	Pv2.1_07_1182132_A/G	Pv07	1,182,132	7.17E−08	0.11	203	0.17	4.15
*WAC3.1*	VEF	Pv2.1_03_51024185_T/A	Pv03	51,024,185	5.56E−10	0.05	330	0.17	9.89
*WAC5.1*	VEF	Pv2.1_05_7726366_C/T	Pv05	7,726,366	1.99E−06	0.03	330	0.12	–8.39
*WAC7.1*	MIP	Pv2.1_07_3412439_T/A	Pv07	3,412,439	6.00E−07	0.12	193	0.20	3.11

*The complete list is presented in Supplementary Tables 5,6.*

*^*a*^Position is based on the *P. vulgaris* reference genome.*

*^*b*^Minor allele frequency.*

*^*c*^R^2^ is the percent of phenotypic variation explained by the SNP marker.*

*^*d*^Allelic effect based on the reference allele.*

CKT3.1 was identified using GWAS in the MAGIC population, confirming the result of the QTL analysis. CKT3.1 has the highest R^2^ value, explaining 34% of the phenotypic variation with an allelic effect of -1.98 min (Tables 3, 4 and Figures 4, 6). Five QTL were identified in the haplotype-based interval mapping, but only CKT3.1 was confirmed by GWAS. WAC7.1 was identified in both Mesoamerican populations, the MAGIC population and the MIP panel (Table 4 and Figure 5). CKT2.1 was identified only in the MIP panel.

### Genomic Prediction Models

**FIGURE 7:**
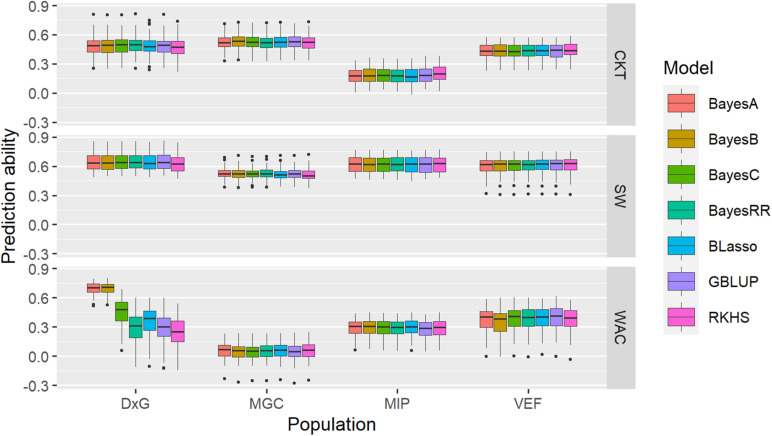
Boxplots of accuracies of cross-validations for genomic predictions for cooking time (CKT), water absorption capacity (WAC), and seed weight (SdW) using different statistical models.

A single-trait assessment of genomic prediction with the CKT, WAC, and SdW data was performed for each population, following optimal custom settings. The overall mean prediction ability (PA) for CKT ranged between 0.18 (MIP) and 0.52 (MAGIC), while the mean PA for WAC ranged between 0.05 (MAGIC) and 0.43 (DxG); the PA for SdW is higher than the other traits, ranging between 0.52 (MAGIC) and 0.64 (DxG) (Figure 7, Supplementary Table 7). In general, the PAs fell significantly below the estimated broad-sense heritability (Supplementary Table 1) and the PAs for WAC were not correlated with the heritabilities. Evaluating the PA of different GP models mostly resulted in very similar results for each trait except for WAC in the DxG. In this case, the PA reached mean values of 0.67 for the BayesA and BayesB priors, doubling the mean PA of other models (0.3) for the same trait in the DxG.

A cross prediction between CKT, WAC, and SdW was performed between and within the MAGIC, MIP, and VEF populations to assess prediction ability using different training-validation datasets. The cross-validation results between traits in the same population followed the behavior of the phenotypic correlations, with PAs ranging between -0.36 (VEF, CKT as training and SdW as validation) and 0.25 (VEF, WAC as training, and SdW as validation) (Table 1 and Supplementary Figure 5). The PA across traits and populations did not show higher values than those obtained in the single-trait assessment of genomic prediction, with PA values ranging from −0.25 (MIP-CKT as training and VEF-SdW as validation) and 0.38 (MIP-SdW and MAGIC-SdW used both as training and validation). GPs across populations for the same trait were only acceptable for SdW between the Mesoamerican populations with PA values of 0.37 and 0.38, but not for other traits.

Taken together, these results show that the PA across populations for CKT, WAC, and SdW is mostly low suggesting different genetic bases. GPs within populations, on the other hand, show promise for breeding applications with acceptable predictive abilities, as long as it would be applied within these genetic groups.

## Discussion

Several factors such as taste, nutrition, cost, and convenience influence the food choice of consumers (Aggarwal et al., 2016). The convenience is defined as a food that is beneficial to the consumer at any of the meal preparation and consumption stages and is exemplified with reductions in time or physical energy, among others. For this reason, the convenience has a significant impact on society’s food consumption behaviors (Ternier, 2010). Cooking time is increasingly recognized as an important trait. Not only do consumers demand products that cook faster to fit a modern lifestyle (Mkanda et al., 2007) but it also affects nutrition and time expenditure and, with the latter, the possibility of women empowerment (Carrigan and Szmigin, 2006). Furthermore, using wood and charcoal as a source of energy for domestic purposes has detrimental effects on the health and environment (Smith, 2006). Obtaining fuel in rural areas can be dangerous and time consuming, or costly in urban areas. We aimed to elucidate the genetic architecture of cooking time in the common bean, a grain legume that takes long preparation times to reach palatability but constitutes an important source of nutrients for millions of people in Latin America and Eastern/Southern Africa. For this purpose, we used germplasm from different breeding panels and genetic populations of the Andean and the Mesoamerican genepools, incorporating a wider genetic variability compared with previous studies.

High genetic variability in all four populations was found, in line with a previous report (Cichy et al., 2019). Also, heritabilities of CKT and WAC were high indicating good data quality for genetic studies, reaching comparable values to previous reports on this trait (> 0.8) (Elia et al., 1997; Jacinto-Hernandez et al., 2003; Arns et al., 2018; Cichy et al., 2019). Some lines that had fast cooking time also have desirable features of grain and agronomic quality (such as seed color and size, high yield, tolerance to drought, and resistance to some diseases, among others) for different market classes (Supplementary Table 2). These lines can be used in the breeding programs to generate new varieties adapted to geographic areas depending on the consumer preferences, contributing to achieve all the benefits that fast-cooking beans can bring for the environment and household habits.

The seed is a living organism that can be susceptible to the processing and manipulation that is carried out right after harvest. Long periods in non-optimal storage conditions have been reported to increase cooking time due to the hard-to-cook (HTC) phenomenon (Coelho et al., 2007; Arruda et al., 2012). The DxG population, which was stored the longest in sub-optimal conditions showed the longest cooking times (Figure 1). The HTC effect was observed here for some samples, where the seed failed to absorb water during the soaking stage, causing an extensive increase in cooking time. The HTC phenomenon causes physical alterations to the cell structure of the seed coat, which reduces the capacity of the grains to absorb water resulting in longer cooking periods (Reyes-Moreno et al., 1993). Sandhu et al. (2018) reported that HTC is an environment-induced phenomenon, but there might be some genetic characteristics of the seed playing a role because some varieties are more prone to the HTC effect than others. For example, Cominelli et al. (2020) found that the genotypes with the low phytic acid 1 (lpa1) mutation were more susceptible to HTC. These findings suggest that HTC may trigger the expression of some genes related to CKT or WAC.

The populations with Andean contribution (DxG population and VEF panel) had a significant negative correlation between WAC and CKT, in parallel with previous reports (Elia et al., 1997; Rodrigues et al., 2005; Cichy et al., 2015; Wani et al., 2017; Berry et al., 2020). During the soaking stage, the water enters the bean through the micropyle migrating below the seed coat, causing a softening effect on the seed as the available water inside the cotyledon allows the cell separation during cooking (Chigwedere et al., 2018). This effect would allow the indirect selection of fast-cooking genotypes through WAC, which is easier, faster, and cheaper to measure. However, in Mesoamerican populations, such correlation was not observed, which may indicate that the genetic mechanism that regulates CKT and WAC is different for each genepool.

In this study, we compared external characteristics of the seed such as weight and color with its cooking time. The correlation between CKT and SdW was not consistent within populations. Some studies have shown weak relationships between CKT and SdW. Cichy et al. (2015) found a positive correlation between these two traits in the Andean Diversity Panel (ADP). However, a parallel study that used a subset of the ADP reported negative correlations between these traits (Katuuramu et al., 2020). This suggests there is no phenotypic or genetic correlation between seed size and time needed to cook it. There was a subtle effect related to the seed color. Seeds with white coats were the fastest cooking group in both the VEF and MIP panels. Similarly, Cichy et al. (2015) found white seeded varieties in the Andean diversity panel (ADP) to be the fastest cooking. On the other hand, red, red-mottled, and cream-mottled beans were the slowest to cook here, resembling the results obtained for the ADP. In this work, we also observed similar trends in the Mesoamerican panel, with white seeded beans cooking the fastest. Although the Mesoamerican black beans in both populations (MAGIC and MIP) were slow cooking, even more so were the yellow lines. These results go in line with the slow cooking yellow Mesoamerican beans reported by Wiesinger et al. (2018). Previous studies have described how low levels of phenols in the seed coat may be correlated with faster cooking time (Hincks and Stanley, 1986; Stanley et al., 1990). Phenol contents are secondary metabolites produced in the cotyledons that can participate in chemical reactions resulting in restricted water binding and impaired cell separation during cooking. Taken together, the seed coat color appears to be related with cooking time, as lighter seeds cooked faster than darker seeds. Nevertheless, cooking times of the color-based groups overlap between each other, so other factors apart from the chemical compounds cause the color to affect cooking properties.

### QTL Results and Use in Breeding

Recently, an increasing number of studies in common bean have investigated the genetics of cooking time; among them, several QTL studies (Jacinto-Hernandez et al., 2003; Vasconcelos et al., 2012; Berry et al., 2020) and studies in the Andean diversity panel (Cichy et al., 2015). However, few studies have focused their results on breeding. Furthermore, the genetic variability analyzed has been limited, focusing on germplasm from a single gene-pool or biparental populations characterized by their limited genetic variability. In this study, we analyzed different representative populations of the two important gene pools existing in common bean: the Andean and Mesoamerican pools (Figure 3).

A QTL was found in populations with Andean contribution (DxG population and VEF panel) with opposite effects on CKT and WAC (CKT3.2/WAC3.1). The favorable allele in DxG is contributed by the Andean parent G19833. This locus likely causes the negative correlation that was observed between CKT and WAC in these populations. A similar QTL was previously described for WAC and CKT in chromosome Pv03 (Pérez-Vega et al., 2010; Berry et al., 2020). WA3 and CT3.1 were identified in a biparental population obtained by crossing the lines Xana (Andean) and Cornell49-242 (Mesoamerican). Similarly, the positive additive effect for WAC originated from the Andean parent, and the closest marker SR20 is located at 50.18 Mbp, not far from WAC3.1 at 51–52 Mbp. These results indicate that WA3 and WAC3.1 are likely the same QTL, which has a reducing effect on CKT in Andean populations. On the contrary, CT3.1 was located in Pv03 but is not in the same position as that of CKT3.2 or CKT3.1 (14–22 Mpb).

The genetic control of WAC may be different in the Andean and Mesoamerican lines investigated here. WAC7.1 was identified in the populations with Mesoamerican origin (MAGIC and MIP panel). The phenotypic correlations between WAC and CKT were distinct, and accordingly, different QTL were identified in this study at chromosomes Pv03 for the Andean and Pv07 for Mesoamerican populations. WAC7.1 co-locates with the *ASP* locus (0–1.5 Mb) associated with seed coat luster: Mature dry black bean seeds are either opaque (dull) or shiny (glossy) (Cichy et al., 2014). The Asp gene is the major determinant of water uptake in black beans. The Asp gene influences the thickness and uniformity of the epicuticular wax layer such that shiny-seeded beans have a thick and more uniform wax layer than opaque-seeded beans. The effect on water uptake is hypothesized to be related to the unevenness of the surface of the opaque beans (Sandhu et al., 2018).

The QTL CKT2.1 and CKT3.1 were identified in the MIP panel and the MAGIC population, respectively. Both QTL were previously reported in the Andean panel ADP (Cichy et al., 2015). In CKT3.1, the founder lines SEN56, INB841, INB827, MIB778, and SXB412 display the desirable negative allelic effects diminishing values for this trait. These five founders were reported to bear introgressions from the Andean genepool at this genomic position (Lobaton et al., 2018). This suggests that alleles of Andean origin contribute to favorable cooking time in these breeding lines.

Several studies have tried to identify the genetic characteristics of CKT and WAC in an effort to unravel their genetic architecture. In all cases, they confirm a relatively high heritability. Some reports indicate that both traits can be controlled by a small number of genes (Elia et al., 1997; Jacinto-Hernandez et al., 2003; Arns et al., 2018), while others indicate that CKT may be under the control of multiple genes at the same time (Vasconcelos et al., 2012; Cichy et al., 2015). The phenotypic variation observed here for those traits support a quantitative mode of inheritance. Even though several QTL were found in this study, the average proportion of explained variance is 23%, reaching a maximum value of 34%. In that sense, an important part of the genetic effects is not captured. It is questionable that these QTL are sufficient to guide a breeding program. None of them were identified across all populations, and potential GxE effects should be studied, though GxE of cooking time has been reported to be limited (Katuuramu et al., 2018; Cichy et al., 2019; Katuuramu et al., 2020).

Given that CKT and WAC appear to have a partially quantitative mode of inheritance, we evaluated to what extent genomic prediction models can capture its genetic variability. Prediction accuracies for CKT ranged from 0.18 to 0.52, suitable for breeding in the MAGIC population, but not so for the MIP panel. Higher accuracies were observed for SdW, ranging from 0.52 to 0.64, close to previously reported values for this trait (Keller et al., 2020). Similarly, higher PAs were reported in common bean for nematode response (Wen et al., 2019). PAs for some agronomic traits were reported to follow the pattern of trait heritabilities, usually ranging 10–20 points below the heritability (Keller et al., 2020). However, this pattern was not observed on individual predictions of CKT or WAC, where PAs were often quite low. Similarly, the PAs in the cross-prediction scenario using different training and validation datasets were even lower than the single-trait prediction scenario. It is not clear at this point why accuracies are not well linked to trait heritabilites as observed in most other cases. We tested several GP models that are based either on additive effects only (Bayes A, B, C, BayesRR, BLasso, and GBLUP) or additive and non-additive effects (semiparametric RKHS) (Figure 7). These results indicate that the genetics of this trait may not be well represented in any of the tested GP models. Even so, the results of prediction ability in some populations seems suitable to be employed in breeding considering that CKT is a complex trait, which allows taking the first steps of genomic prediction and genomic selection in breeding programs focused on seed quality.

In this work, we compared different population types, using constructed bi-parental and eight-parental RIL populations besides two different breeding panels. All population types appear basically suitable for identifying genetic variability, for genetic mapping, and GP. RIL populations performed somewhat better for predicting CKT and WAC. This was not observed in studies with other traits comparing GP in MAGIC population and VEF panel (Keller et al., 2020). Panels of elite breeding lines provide more relevant variability that can be directly applied in germplasm improvement; hence, this information is more valuable for breeders.

## Conclusion

This study evaluated the genetic architecture of cooking time and water absorption capacity using and integrating different tools and methodologies. To our knowledge, this study used the highest genetic variability studied so far in these traits in common bean, using four different populations with lines belonging to both Andean and Mesoamerican gene-pools. The presented results validate the advantage of combining GWAS and QTL analyses to find loci that controlled a complex trait. We identified fast cooking lines in every population evaluated with a high potential to be implemented in a breeding program with perspectives to different markets. Different QTL for the Andean and Mesoamerican gene-pool were located in distinct regions of the genome, suggesting differential genetic control in each of the pools for the traits of interest. Genomic selection looks to be a promising tool in several of the evaluated populations; offspring populations need to be evaluated to see if the understanding of variation in accuracy can be improved in the future. Genomic selection is particularly promising if the investment for genotyping can be used to predict several traits at a time, in which case also a lower accuracy trait can be added to a selection index.

## Data Availability Statement

The SNP marker matrices, the raw and modeled phenotypic data, and genetic maps used in this study are available for download at Harvard Dataverse: https://doi.org/10.7910/DVN/B3YLRF. Data presented in other studies: https://doi.org/10.7910/DVN/XCD67U and https://doi.org/10.7910/DVN/JR4X4C, as published by Diaz et al. (2020) and Keller et al. (2020).

## Author Contributions

SD, RR, NA, and CH carried out the field trial evaluations. SD, DA-S, JA, RR, H-PP, and BR did the phenotypic data analysis. HD and HR developed the hardware and software systems to measure cooking time. SD, DA-S, and RR performed the genotypic data analysis. SD and DA-S did the association, linkage analysis, and genomic prediction models. SD, DA-S, and BR wrote the manuscript. All authors read, contributed to, and approved the final version of the manuscript.

## Conflict of Interest

The authors declare that the research was conducted in the absence of any commercial or financial relationships that could be construed as a potential conflict of interest.

## Footnotes

1 https://hudsonalpha.org/

2 https://www.biotech.cornell.edu/core-facilities-brc/services

## Supplementary Material

Supplementary Figure 1 Crossing scheme of the MAGIC population.

Supplementary Figure 2 Detailed scheme about methods applied for each population and materials available for each analysis.

Supplementary Figure 3 Genetic map of DOR364 x G19833 (DxG) population, using 561 markers mapped to 11 linkage groups. The map was developed with the Kosambi mapping function using MapDisto Software (v1.7) (Lorieux, 2012).

Supplementary Figure 4 Location of QTL *CKT3.2* and *WAC3.1* and their corresponding LOD values on chromosome Pv03 in the genetic map of the DOR364 x G19833 population.

Supplementary Figure 5 Correlogram with the results of the cross-prediction scenario using different training (X axis) and validation (Y axis) datasets. The elements of the broad diagonal (white text) show the mean prediction ability within each population from 100-fold cross validation partitions. The values in square brackets show the phenotypic correlation between each pair of traits. The off-diagonal elements (black text) show the prediction accuracy from a single training-validation step between traits of different populations.

Supplementary Table 1 Phenotypic variation, and broad-sense heritability (H^2^) for cooking time (CKT), water absorption capacity (WAC) and 28 seed weight (28SdW) evaluated in four populations: DxG RIL population and 2 parents, MAGIC population and 8 founders, VEF panel and MIP panel.

Supplementary Table 2 The 5% of the fastest cooking lines for DxG population, MAGIC population, VEF panel and MIP panel.

Supplementary Table 3 Genetic map of the MAGIC population using 5739 markers mapped to 11 chromosomes. The map was developed using haplotype-based interval mapping (Diaz et al., 2020).

Supplementary Table 4 Identified QTL for cooking time (CKT) and water absorption capacity (WAC) in the MAGIC population based on haplotype prediction using composite interval mapping with the procedure for additive effects (ICIM-ADD). The additive effect and mean for the haplotype’s groups are shown.

Supplementary Table 5 Complete GWAS results for all markers used in association analyses for cooking time, evaluating the MAGIC population, VEF panel and MIP panel.

Supplementary Table 6 Complete GWAS results for all markers used in association analyses for water absorption capacity, evaluating the MAGIC population, VEF panel and MIP panel.

Supplementary Table 7 Genomic predictions for cooking time (CKT) and water absorption capacity (WAC) using different statistical models in four populations.

Supplementary Document 1 Hardware and software design of cooking time measure machine.
