# Supplementary figures and images for: Genetic Architecture and Genomic Prediction of Cooking Time in Common Bean (Phaseolus vulgaris L.)
Source: Front Plant Sci. 2021 Feb 11;11:622213. doi: 10.3389/fpls.2020.622213 (PMC7905357; doi:10.3389/fpls.2020.622213)

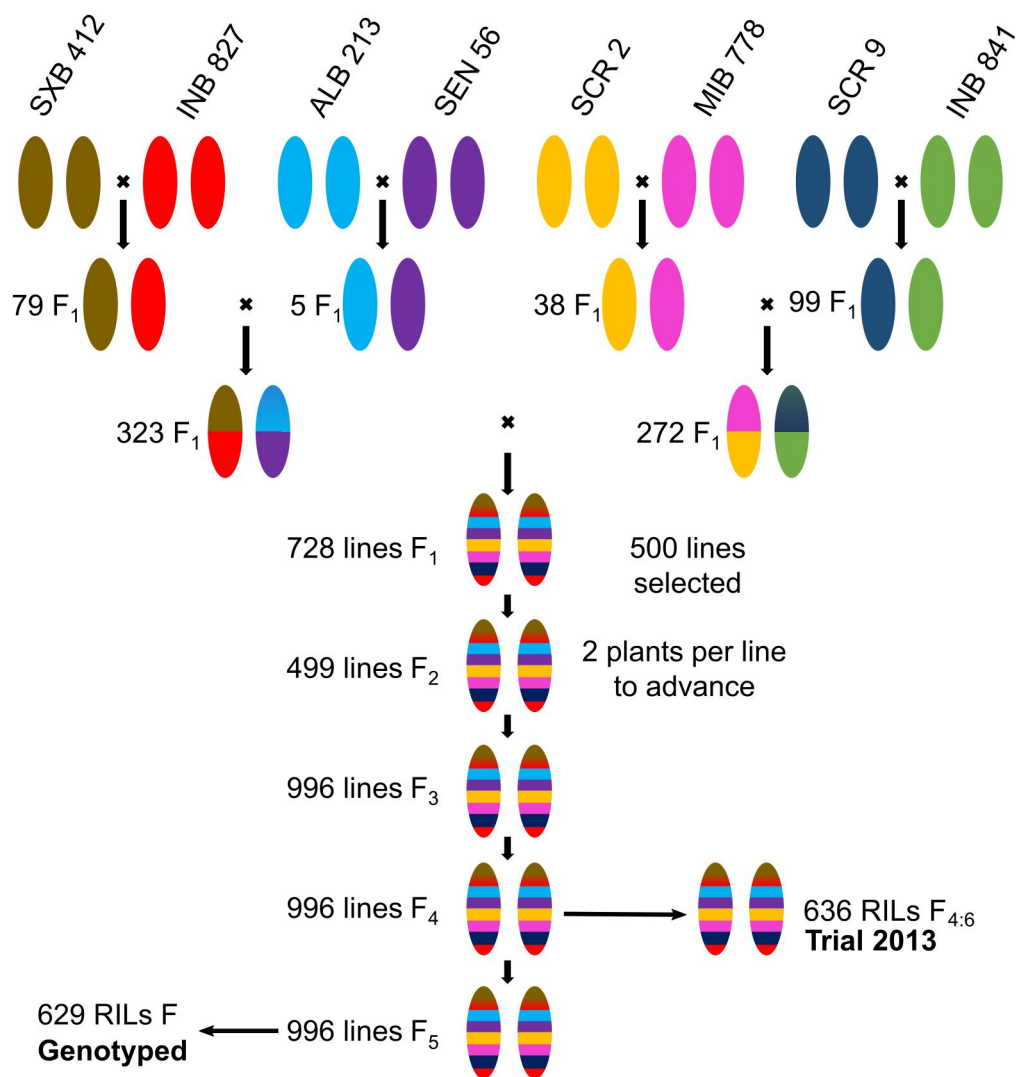

Supplement: Supplementary Figure 1 — Crossing scheme of the MAGIC population. [file Image_1.PDF]

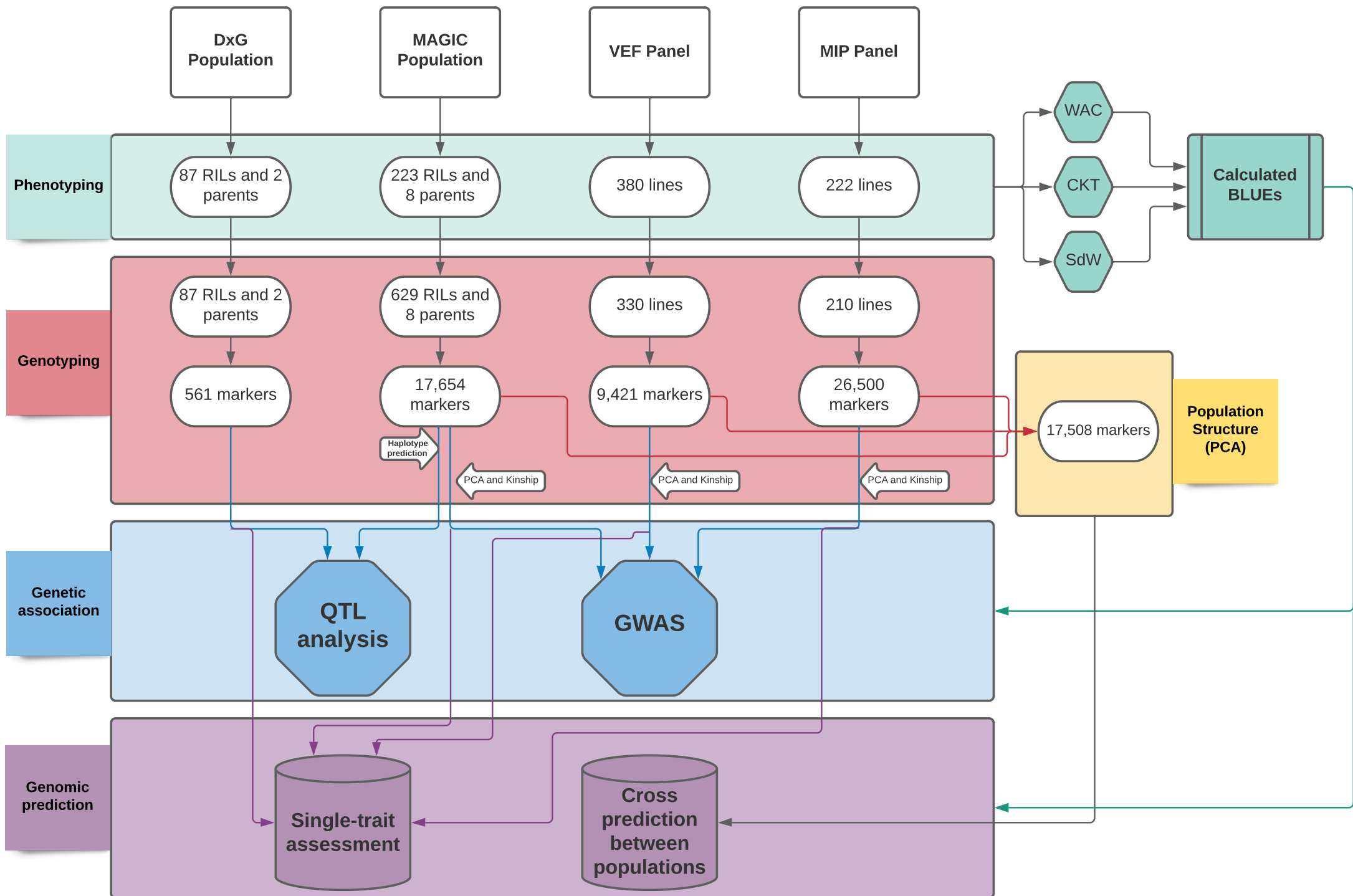

Supplement: Supplementary Figure 2 — Detailed scheme about methods applied for each population and materials available for each analysis. [file Image_2.PDF]

# Pv01

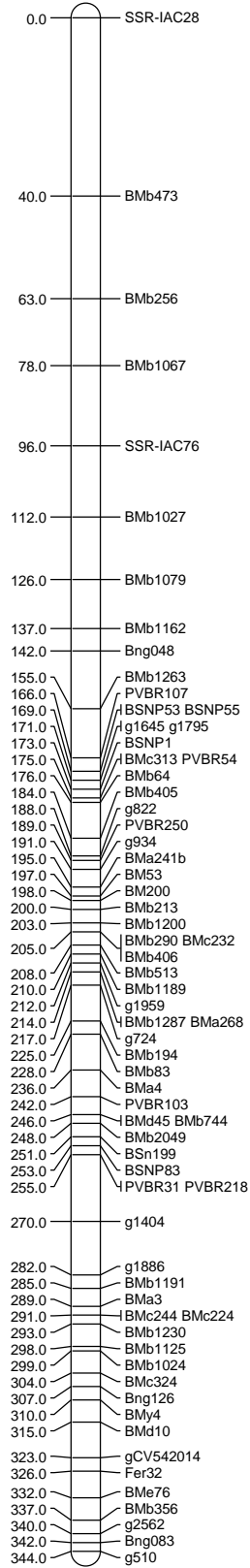

# Pv02

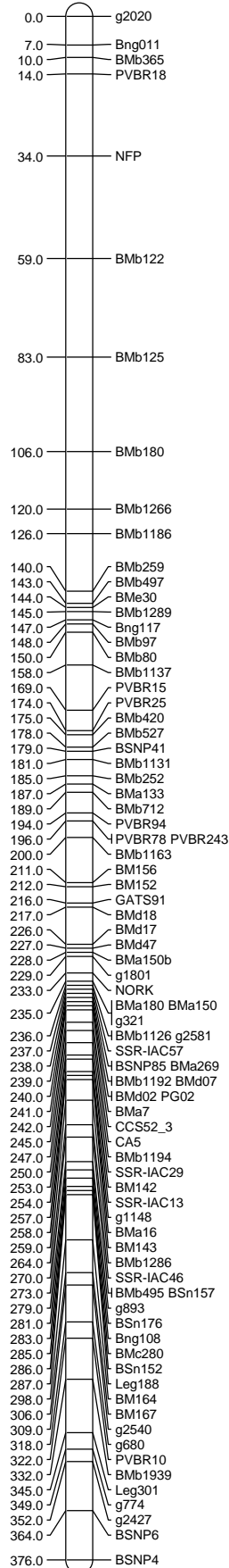

# Pv03

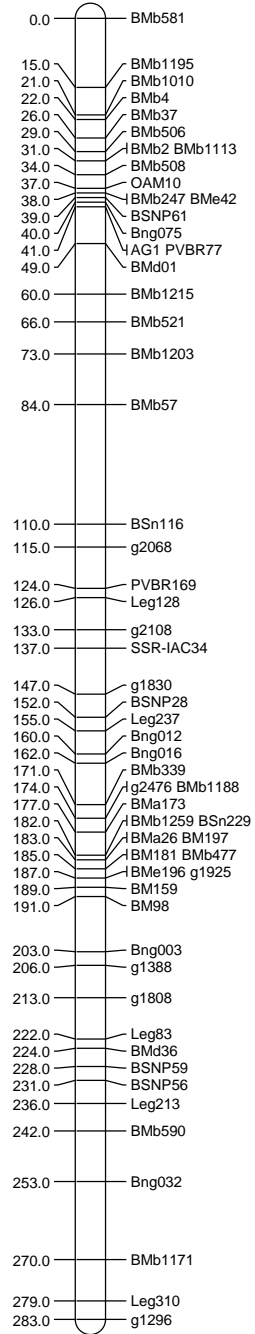

# Pv04

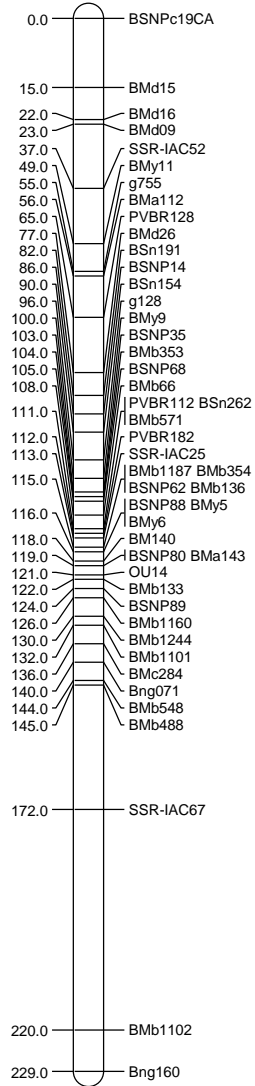

# Pv05

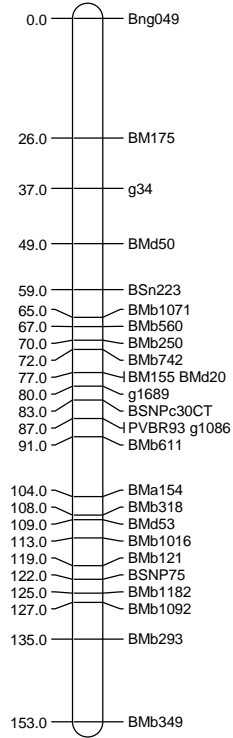

# Pv06

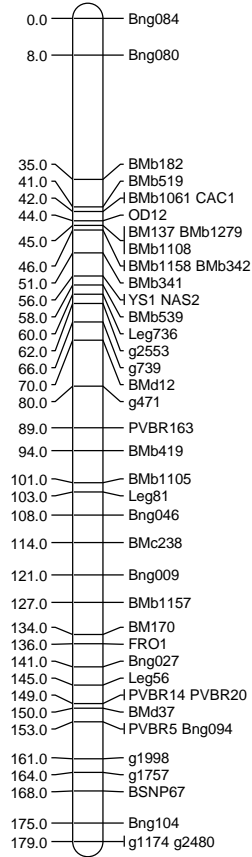

# Pv07

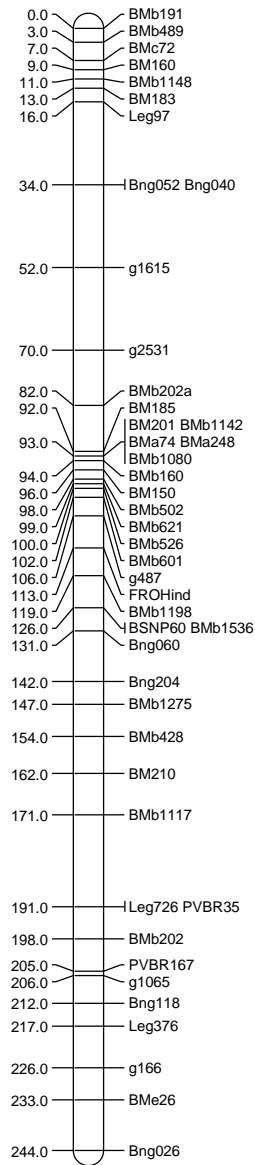

# Pv08

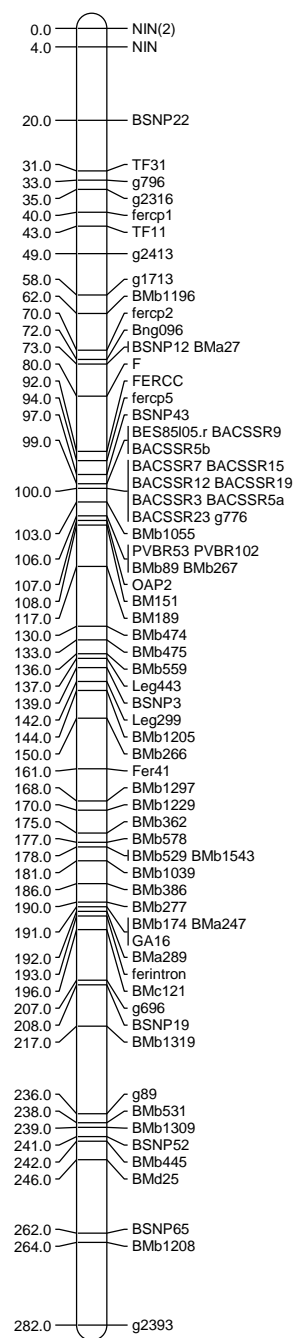

# Pv09

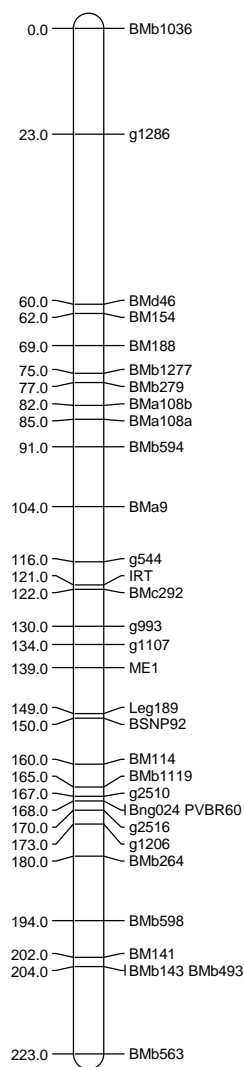

# Pv10

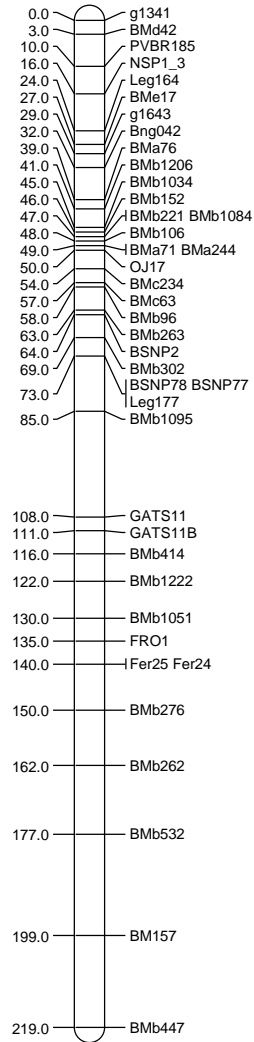

# Pv11

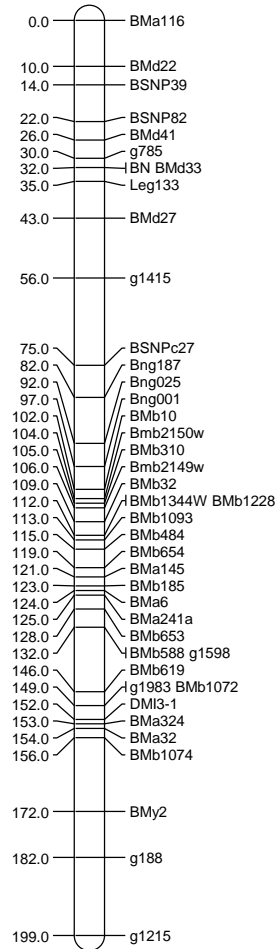

Supplement: Supplementary Figure 3 — Genetic map of DOR364 x G19833 (DxG) population, using 561 markers mapped to 11 linkage groups. The map was developed with the Kosambi mapping function using MapDisto Software (v1.7) (Lorieux, 2012). [file Image_3.PDF]

# Chr3

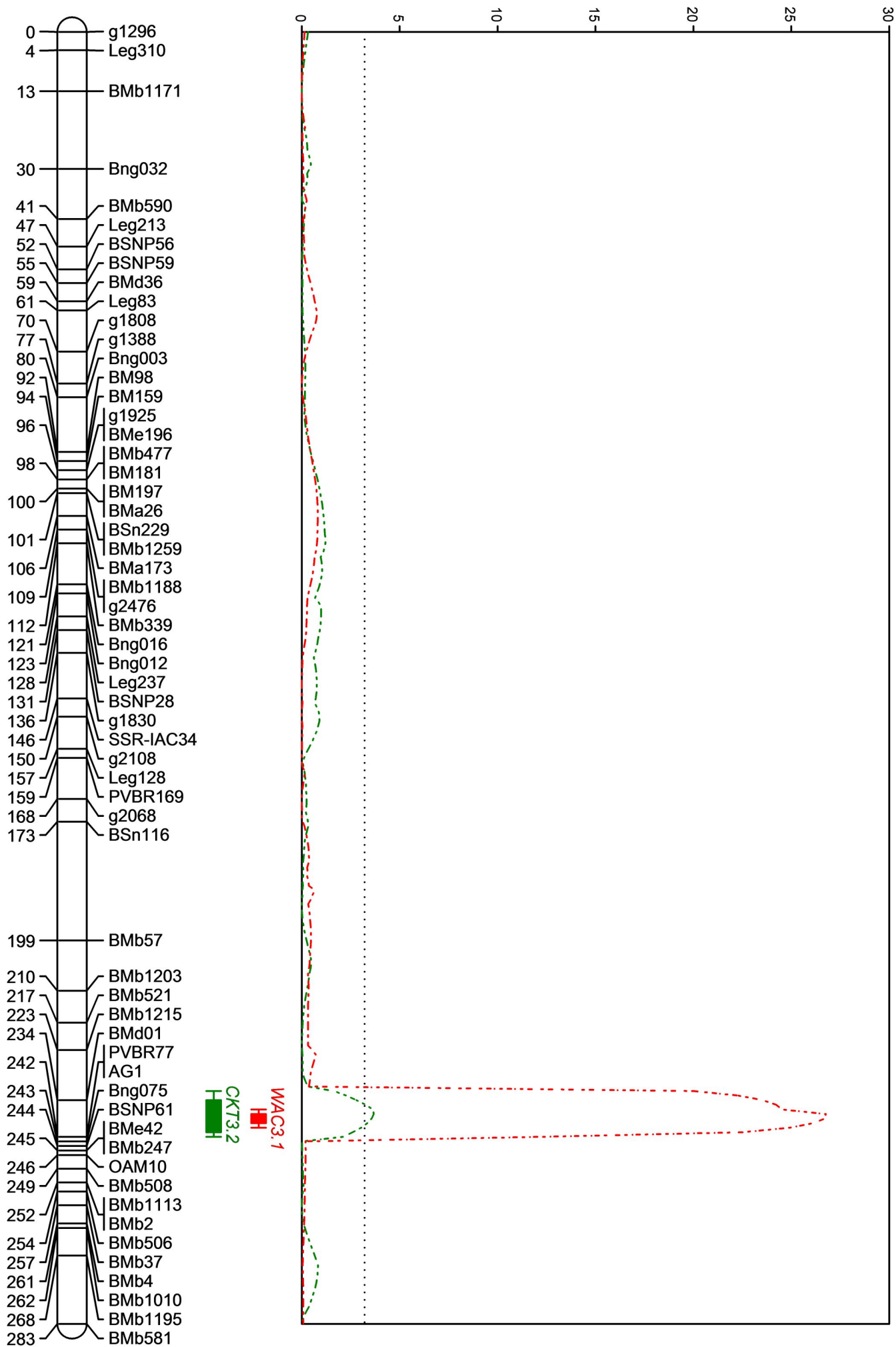

Supplement: Supplementary Figure 4 — Location of QTL CKT3.2 and WAC3.1 and their corresponding LOD values on chromosome Pv03 in the genetic map of the DOR364 x G19833 population. [file Image_4.PDF]
