# Supplementary material for: Genetic Architecture and Genomic Prediction of Cooking Time in Common Bean (Phaseolus vulgaris L.)
Source: Front Plant Sci. 2021 Feb 11;11:622213. doi: 10.3389/fpls.2020.622213 (PMC7905357; doi:10.3389/fpls.2020.622213)
Supplement: Supplementary Figure 5 — Correlogram with the results of the cross-prediction scenario using different training (X axis) and validation (Y axis) datasets. The elements of the broad diagonal (white text) show the mean prediction ability within each population from 100-fold cross validation partitions. The values in square brackets show the phenotypic correlation between each pair of traits. The off-diagonal elements (black text) show the prediction accuracy from a single training-validation step between traits of different populations. [file Image_5.PDF]

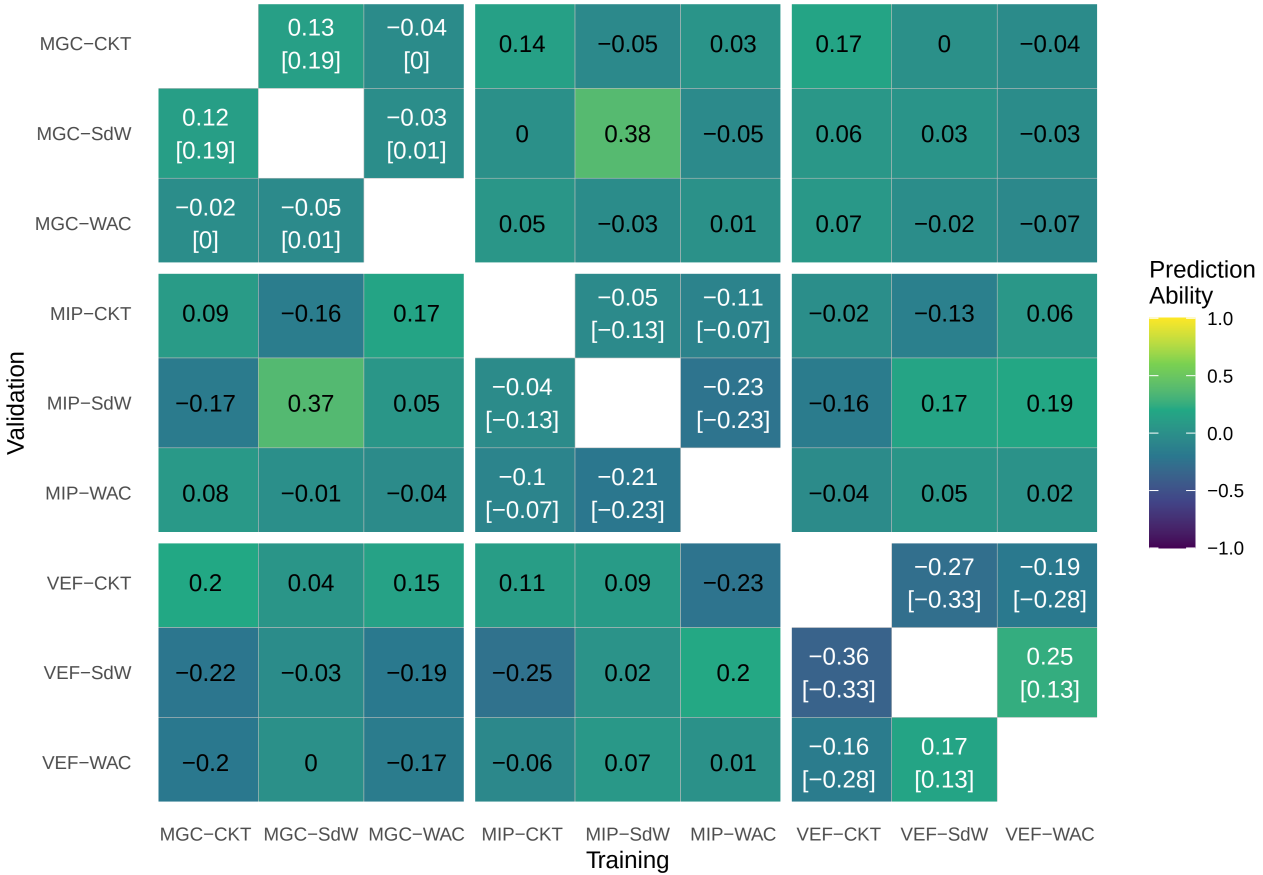

Validation
Training
Prediction Ability
1.0
0.5
0.0
−0.5
−1.0
MGC−CKT
MGC−SdW
MGC−WAC
MIP−CKT
MIP−SdW
MIP−WAC
VEF−CKT
VEF−SdW
VEF−WAC
0.13 [0.19]
−0.04 [0]
0.14
−0.05
0.03
0.17
0
−0.04
0.12 [0.19]
−0.03 [0.01]
0
0.38
−0.05
0.06
0.03
−0.03
−0.02 [0]
−0.05 [0.01]
0.05
−0.03
0.01
0.07
−0.02
−0.07
0.09
−0.16
0.17
−0.05 [−0.13]
−0.11 [−0.07]
−0.02
−0.13
0.06
−0.17
0.37
0.05
−0.04 [−0.13]
−0.23 [−0.23]
−0.16
0.17
0.19
0.08
−0.01
−0.04
−0.1 [−0.07]
−0.21 [−0.23]
−0.04
0.05
0.02
0.2
0.04
0.15
0.11
0.09
−0.23
−0.27 [−0.33]
−0.19 [−0.28]
−0.22
−0.03
−0.19
−0.25
0.02
0.2
−0.36 [−0.33]
0.25 [0.13]
−0.2
0
−0.17
−0.06
0.07
0.01
−0.16 [−0.28]
0.17 [0.13]
