# Supplementary material for: Genetic Architecture and Genomic Prediction of Cooking Time in Common Bean (Phaseolus vulgaris L.)
Source: Front Plant Sci. 2021 Feb 11;11:622213. doi: 10.3389/fpls.2020.622213 (PMC7905357; doi:10.3389/fpls.2020.622213)
Supplement: Supplementary Document 1 — Hardware and software design of cooking time measure machine. [file Data_Sheet_1.DOCX]

**A friendly, low-cost and open-source automated Mattson-based cooker for determining of cooking times of common beans**

The research team at CIAT-HQ in the Bean Program, modified the Mattson cooker apparatus to become an automated Mattson-based cooker (Fig.1) using an embedded system for taking data from each seed individually. This automation (Fig.2) consists of a set of disk plates and screw rods fixer, stainless steel plunger with a 90g of weight and 2mm pin, custom-made printed circuit board assembly, ribbon cables, router for Wi-Fi communication, Udoo system and power supply. Furthermore, a PT100 sensor was added to allow monitoring the temperature throughout the experiment. Finally, a web application was developed to monitor and control wirelessly the process on any computer or mobile device (Fig.3).

Advantages:

- 2 independent processes running at the same time.
- All data is automatically recorded into the database.
- It does not require permanent attention.
- Events saved every time plunger drops, even if happens simultaneously.
- Friendly user interface.
- Small and compact size.
- Wireless communication.
- Allows mobile devices to control the process.
- Open-source and low-cost efficient resources.


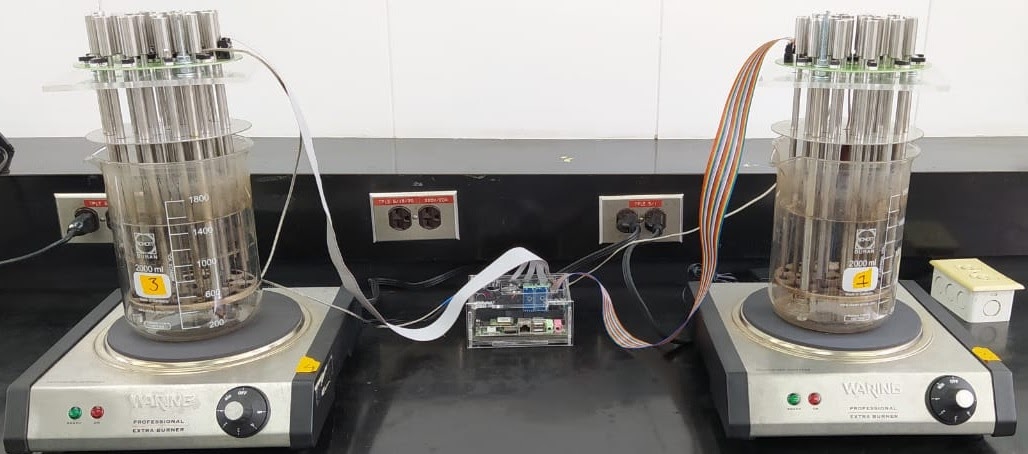


Fig.1


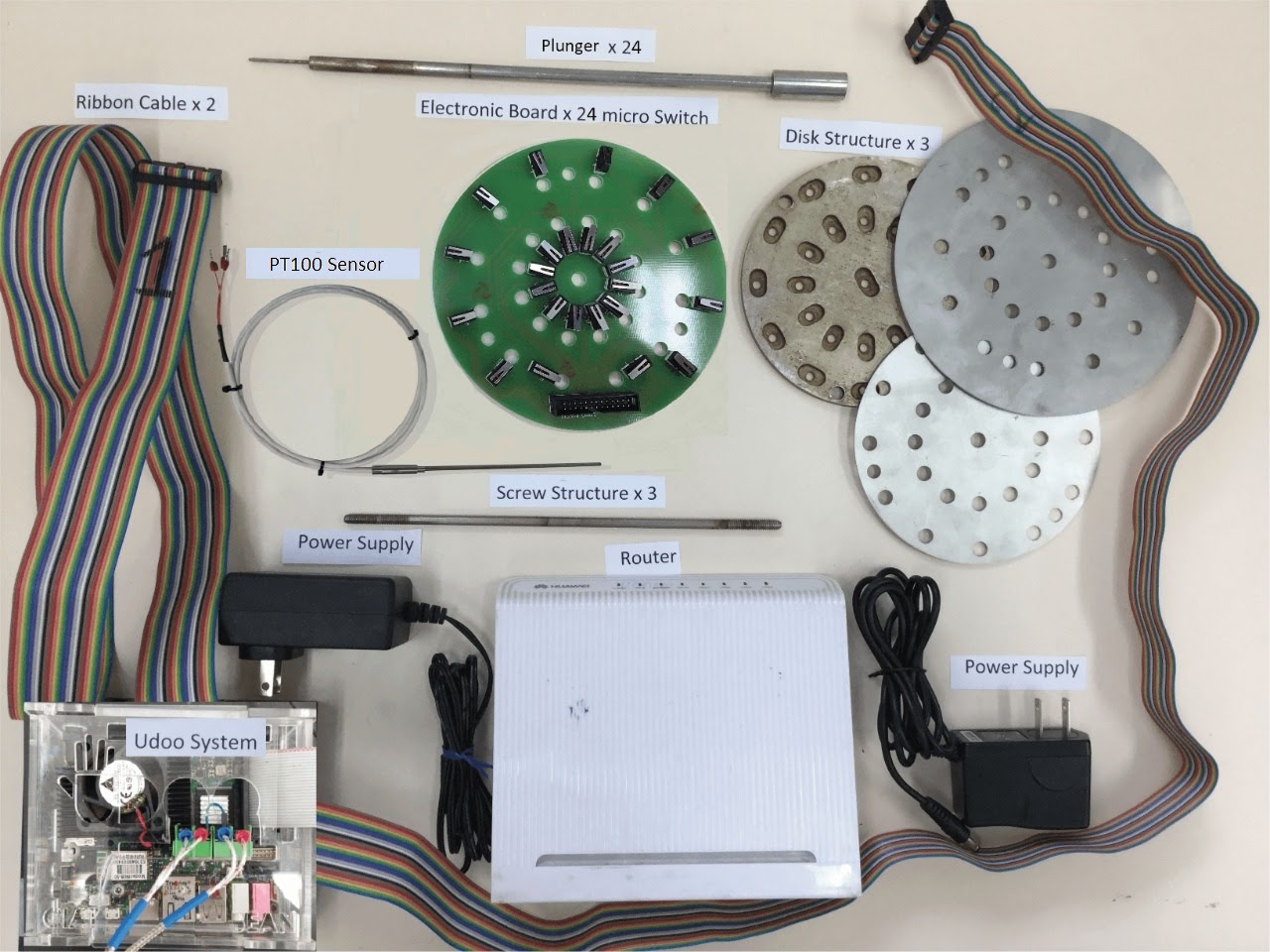


Fig.2


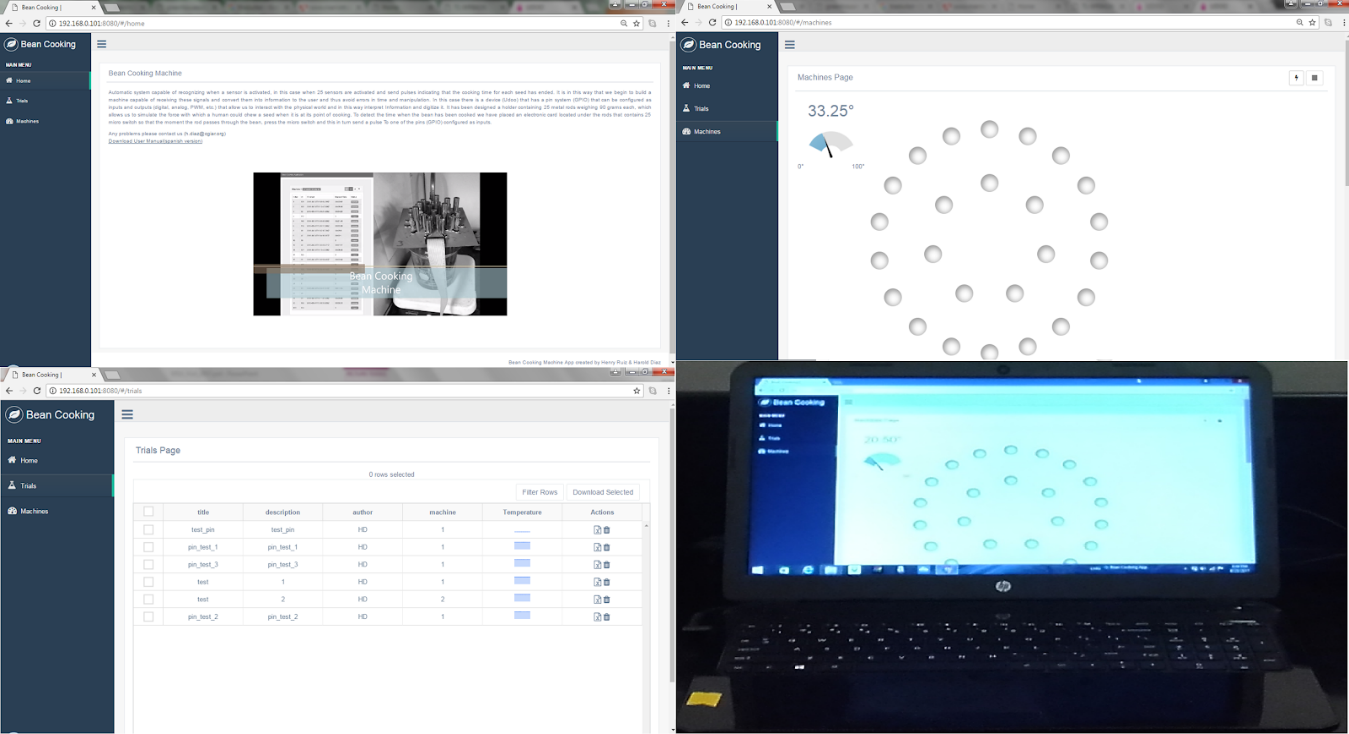


Fig.3

Udoo system: <https://www.udoo.org/udoo-dual-and-quad/>

NodeJS: <https://nodejs.org/>

a)    The udoo system allows 48 GPIO pins to monitor the process, two Mattson cooker modified devices simultaneously.

b)    The circuit is designed for connecting 24 switches to the udoo system.

c)    The udoo system receives the signal level from the circuit board.

d)    The switch is OFF, when the plunger is located in a high position and the logic level is zero.

e)    The switch is ON, when the plunger drops down and the logic level is one.

f)     At the beginning, all 48 pins switches are in the OFF position.

g)    When the plunger penetrates the seed during the cooking process and drops a short distance, the switch is actioned.

h)    The time when each plunger actuates the switch is recorded through a web application software developed using NodeJS approach.
